# Development of a Modelling to Correlate Site and Diameter of Brain Metastases with Hippocampal Sparing Using Volumetric Modulated Arc Therapy

**DOI:** 10.1155/2013/568597

**Published:** 2013-10-09

**Authors:** Silvia Chiesa, Mario Balducci, Luigi Azario, Simona Gaudino, Francesco Cellini, Gian Carlo Mattiucci, Cesare Colosimo, Vincenzo Valentini

**Affiliations:** ^1^Department of Radiation Oncology, Università Cattolica del Sacro Cuore, Largo Agostino Gemelli, 8, 00168 Roma, Italy; ^2^Department of Medical Physics, Università Cattolica del Sacro Cuore, Largo Agostino Gemelli, 8, 00168 Roma, Italy; ^3^Department of Radiology, Università Cattolica del Sacro Cuore, Largo Agostino Gemelli, 8, 00168 Roma, Italy; ^4^Department of Radiation Oncology, Campus Bio-Medico, 00155 Roma, Italy

## Abstract

*Purpose*. To correlate site and diameter of brain metastases with hippocampal sparing in patients treated by RapidArc (RA) technique on whole brain with simultaneously integrated boost (SIB). *Methods and Materials*. An RA plan was calculated for brain metastases of 1-2-3 cm of diameter. The whole brain dose was 32.25 Gy (15 fractions), and SIB doses to brain metastases were 63 Gy (2 and 3 cm) or 70.8 Gy (1 cm). Plans were optimized and evaluated for conformity, target coverage, prescription isodose to target volume, homogeneity index, and hippocampal sparing. *Results*. Fifteen brain lesions and RA plan were generated. Hippocampal volume was 4.09 cm^3^, and hippocampal avoidance volume was 17.50 cm^3^. Related to site of metastases, the mean hippocampal dose was 9.68 Gy^2^ for occipital lobe, 10.56 Gy^2^ for frontal lobe, 10.56 Gy^2^ for parietal lobe, 10.94 Gy^2^ for deep brain structures, and 40.44 Gy^2^ for temporal lobe. The mean hippocampal dose was 9.45 Gy^2^, 10.15 Gy^2^, and 11.70 Gy^2^ for diameter's metastases of 1.2 and 3 cm, respectively, excluding results relative to temporal brain lesions. *Conclusions*. Location more than size of metastases can adversely influence the hippocampus sparing. Further investigation is necessary to meet definitive considerations.

## 1. Introduction

Brain metastases are the most common intracranial tumor in adults [1]. Current treatment modalities include whole brain radiotherapy (WBRT), surgery, stereotactic radiosurgery (SRS), and chemotherapy. Whole brain radiotherapy is usually the primary treatment option for patients with multiple brain metastases, while its role remains discussed in oligometastatic patients [2]. 

Many studies reported serious and permanent late side effects after WBRT [3, 4]. 

Recent clinical studies suggest that deficits in learning, memory, and spatial information processing are related to hippocampal damage [5].

Monje et al. hypothesized that memory function is associated with the pyramidal and granule cells located in the dentate gyrus of the hippocampus [6].

Modest radiation doses cause apoptosis decline in neurogenesis in the subgranular zone and then the extinction of short-term memory [7–9].

On the other hand Li et al. [10] observed that tumor shrinkage was significantly correlated with preservation of executive function and fine motor coordination.

Ghia et al. [11] analyzed the distribution of brain metastases and observed that only 3.3% of intracranial metastases were located within 5 mm of the hippocampus; they concluded that it is reasonable to exclude this structure from WBRT when it is not involved by disease.

Modern IMRT techniques allow to deliver highly conformal dose distribution. 

Several authors tested the feasibility of treating the whole brain to full dose sparing the hippocampus, using helical tomotherapy or volumetric modulated arc therapy by LINAC, (RA or VMAT) [12]. But given the complexity of the limbic circuit and the difficulty in delineating the hippocampus on cross-sectional imaging, the peculiar features of treatment plan optimization, and physics quality assurance, this treatment may be complex and time consuming and may need of expertise. 

Primary endpoint of this study was to investigate the impact of location and size of brain metastases on the feasibility of reducing the mean hippocampal dose using VMAT-RA technique; secondary endpoint was to verify if our mean hippocampal dose was close to that obtained by other investigators. 

## 2. Methods and Materials 

A model of 3 metastatic lesions for each lobe, placed in the centre of the lobe, of 1-2-3 cm of diameter was developed. A total of 15 lesions were contoured using a CT simulation scan of an adult patient. 

### 2.1. Acquisition Image and Fusion

Patient was positioned supine in a custom-made mask and underwent a noncontrast CT simulation scan of the entire head region with 1.25 mm slice thickness (1.00 mm slice by reconstruction). The patient underwent three-dimensional spoiled gradient axial magnetic resonance imaging (MRI) scans (3D-SPGR), with standard axial and coronal fluid attenuation recovery (FLAIR), axial T2-weighted and gadolinium contrast-enhanced T1-weighted sequence acquisitions with a 1.00 mm slice thickness.

The CT images were coregistered to a gadolinium-enhanced, T1 weighted, and magnetization preparer rapid gradient-echo axial RM.

### 2.2. Contouring

Anatomic structures were delineated on the coregistered CT-MRI axial image sets using Varian Eclipse External Beam Planning System, version 8.9 (Varian Medical Systems). 

Hippocampus, brain lobes, and deep brain structures were contoured manually with a neuroradiologist on T1-weighted MRI axial sequences according to several atlas of neuroanatomy and to guidelines [12, 13]. 

The hippocampus was contoured on T1-weighted MRI axial sequences, giving the preponderance of gray matter in the hippocampus, contouring focused on the T1-hypointense signal medial to the temporal horn and distinct from the T1-hyperintense parahippocampal gyrus and fimbriae, located inferomedial and superomedial to the hippocampus, respectively. Contouring began at the most caudal extent of the crescentic-shaped floor of the temporal horn and continued posterocranially along the medial edge of the temporal horn. The medial border of the hippocampus was delineated by the edge of the T1-hypointensity up to the ambient cistern. The uncal recess of the temporal horn served to distinguish the hippocampus from the gray matter of the amygdala, lying anterior and superior to the hippocampus. The postero-cranial extent of the hippocampus was defined by the curvilinear T1-hypointense hippocampal tail located just antero-medially to the atrium of the lateral ventricle. Contours terminated at the lateral edges of the quadrigeminal cisterns, before the emergence of the crus of the fornix. 

Brain lobes and critical deep structures included thalamus and basal ganglia, and a single volume, separated from the lobe volumes, was generated. 

The organs at risk included the eyes (whole globe and separate lenses' eye), brainstem, optic nerves, optic chiasm, and hippocampus.

**Figure 1:**
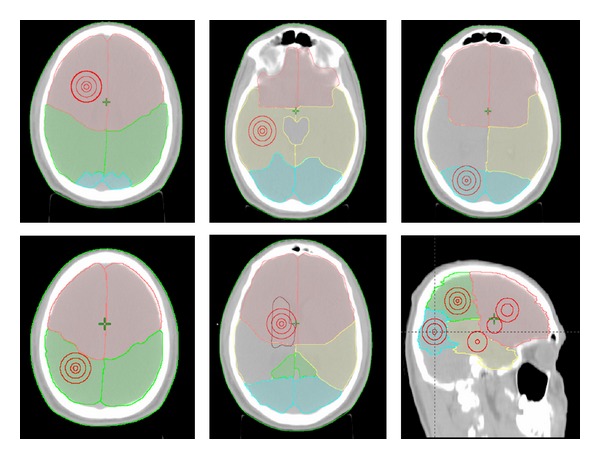
Concentric metastatic lesions in brain's lobes: frontal (pink), parietal (green), temporal (yellow), occipital (cyan), and deep brain structures (brown).

Metastatic lesions were adapted to a spheroid as the metastases' shape. They were drawn in brain lobes and in the deep brain structures. The centre of these spheroids was allocated in the gravity centre of each lobe and deep brain parenchyma; it is considered a geometric representative point of whole lobe's volume. This was obtained by simulating a 3D conformal radiotherapy plan in which the target volume was represented by the lobe or deep brain contoured. On the coordinates *x*, *y*, *z* of the gravity centre, we contoured by brush tool circles of 1-2-3 cm of diameter. These diameters were selected according to the inclusion criteria for stereotactic therapy. Using Pythagoras' theorem we created on up and down slice, a correspondent circumference until to obtain the spheroid with selected diameter; they represented the gross tumor volume (GTV) (Figure 1). 

A 5 mm volumetric margin expansion was applied to the hippocampus. 

A planning target volume for each metastasis (PTV_mts_) was outlined using a computer-automated 2 mm 3D margin expansion. The PTV_mts_ was used for concomitant integrated boost. A whole brain planning target volume (PTV_WB_) was generated by subtracting the PTV_mts_ and the hippocampus avoidance structure from the whole brain contour.

### 2.3. Planning

The prescription doses were 32.25 Gy to 95% of the volume of PTV_WB_, 63 Gy to 95% of the volume of metastases with diameter of 2.0 and 3.0 cm, and 70.8 Gy to 95% of the volume with diameter of 1.0 cm; the dose was delivered in 15 fractions. We used the schedule of Hsu et al. [14] to compare our level of dose to hippocampus using the same technique.

**Figure 2:**
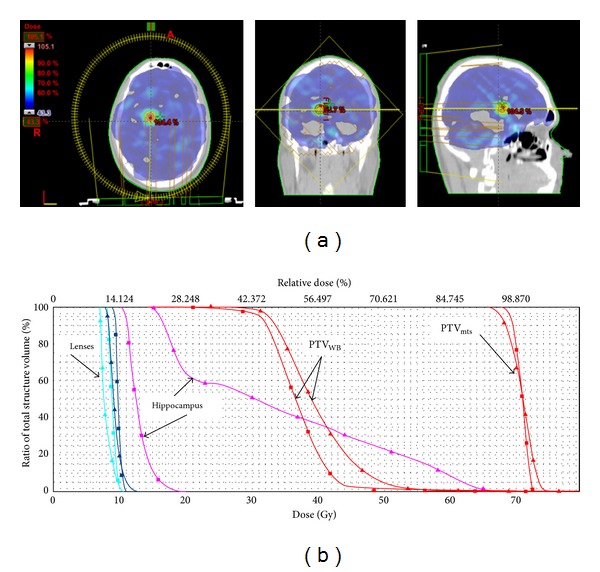
(a) Example of dose distribution of avoidance of hippocampus during whole brain radiotherapy with simultaneously integrated boost using Volumetric Modulated Arc Therapy. (b) Plan comparison cumulative, normalized dose-volume histogram (▲ temporal versus ■ occipital): hippocampus (pink), eyes (cyan/blue), and PTV_mts_ and PTV_wb_ shown. Two metastases of 1 cm of diameter prescribed to 70.8 Gy and whole brain to 32.25 Gy in 15 fractions.

Dose calculations for RA optimization were performed using Varian Eclipse external Beam Planning System, version 8.9 with the AAA algorithm for dose calculation with PRO2 optimization system. The RA plans consisted of a single arc, starting at a gantry angle of 179 and rotating counterclockwise through 358 to stop at a gantry angle of 181. The falloff was 0.2. During optimization multileaf collimator (MLC)-shaped fields are progressively added throughout the arc. The gantry rotation speed and monitor units (MU) per gantry angle degree were optimized for a variable dose rate plan with a maximum dose rate of 400 MU/min, and the nominal energy of photons was 6 MV. During planning, the user defines the prescription dose to the target structures and also the dose constraints to the organs at risk. Step by step for each side and size of metastases we tried to reduce the mean dose for the hippocampus without compromising on coverage of the metastases and whole brain (Figure 2). 

### 2.4. Treatment Planning Evaluation

The treatment plans were evaluated for the following.

#### 2.4.1. Max and Mean Hippocampal Dose

Quantifies the maximal and the mean dose of combined hippocampal volumes; doses were converted to biologically equivalent doses in 2-Gy fractions (Gy^2^), assuming an  *α*/*β* ratio of 2Gy.

#### 2.4.2. Target Coverage (TC)

Describes the fraction of the target volume (*V*_*T*_) receiving at least the prescription dose (*V*_*T* presc_) and is defined as

(1) $$\begin{array}{c} \text{TC}=\frac{V_{T\;\text{presc}}}{V_{T}}. \end{array}$$

According to RTOG QA guidelines, it should be kept close to 1.0. 

#### 2.4.3. Homogeneity Index (HI)

Is defined as the maximum dose delivered to 2% of the target volume (*D*2%) minus the dose delivered to 98% of the target volume (*D*98%) divided by the median dose (*D*_median_) to the target volume:

(2) $$\begin{array}{c} \text{HI}=\frac{\left(D2\%-D98\%\right)}{D_{\text{median}}}; \end{array}$$

it quantifies homogeneity dose distribution in the target volumes. 

#### 2.4.4. V95

Quantifies the volume of PTV_WB_ receiving 95% of the prescription dose or more.

#### 2.4.5. V105

Quantifies the volume of the PTV_WB_ receiving 105% of the prescription dose or more. 

#### 2.4.6. Mean Normalized Tissue Dose (NTD_mean_)

It is defined as the total dose that would have the same biological effect as the actual treatment schedule if it were given in 2-Gy fractions. This parameter allows us to compare the effects on normal tissue for two dose volume histograms. An *α*/*β* ratio of 2 Gy was assumed for the hippocampus and 3 Gy for the eyes:

(3) $$\begin{array}{c} \text{NT}{\text{D}}_{\text{mean}}:\;\frac{\text{Td}\left(\text{Fd}\;+\alpha /\beta \right)}{\left(2+\alpha /\beta \right)}, \end{array}$$

where Td = total dose, Fd = dose for fraction. 

## 3. Results and Discussion

We developed a model using 15 lesions with a diameter included between 1 and 3 cm to support the indication to stereotactic radiosurgery. We contoured 4 lobes (1 frontal, 1 parietal, 1 temporal, 1 occipital lobe), and 1 area of deep brain structures, and hippocampus for each hemisphere. Fifteen brain lesions for each hemisphere were obtained: 3 for each of four lobes and 3 for deep brain critical structure. The GTV_mts_ and PTV_mts_ for diameter of 1-2 and 3 cm were 0.51–1.47 cm^3^, 4.17–7.42 cm^3^, and 14.11–21 cm^3^, respectively. Hippocampal volume was 4.09 cm^3^ and hippocampal avoidance volume was 17.50 cm^3.^

Fifteen RA treatment plans were calculated. Evaluation parameters for each plan are reported in Table 1. 

The mean TC was for PTV_WB_ and PTV_mts_ was 0.9 ± 0.04 and 0.52 ± 0.06, respectively.

Homogeneity for PTV_WB_ was 0.65 ± 0.18. 

The mean hippocampal dose obtained in all RA plans is 10.43 Gy^2^. According to the site of lesion, the mean hippocampal doses, normalized to EQD2, was 9.68 Gy^2^ for occipital lobe, 10.56 Gy^2^ for frontal lobe, 10.56 Gy^2^ for parietal lobe, 10.94 Gy^2^ for deep brain structures, and 40.44 Gy^2^ for temporal lobe. According to the diameter of the lesion, excluding results relative to temporal brain lesions, the mean hippocampal dose, normalized to EQD2, was 9.45, 10.15, and 11.70 Gy^2^ for 1, 2, and 3 cm metastases, respectively. 

Whole brain radiotherapy remains a standard treatment for intracranial brain metastases, but patients might experience neurocognitive toxicity, correlated to effects on the limbic system [6, 7].

Ghia et al. [11] demonstrated that hippocampus can be spared because metastases are generally more than 5 mm away.

Accurate delineation of the hippocampus and its central location are two critical aspects to obtain a good intracranial control of disease without neurocognitive decline. 

Integrated plans of WBRT and SIB for brain metastases have previously been already described, and several authors evaluated the dosimetric feasibility of sparing hippocampus using not volumetric IMRT technique or helical tomotherapy or VMAT [15]. 

At our knowledge this is the first study in which a model has been created to predict the impact of site and size of metastases on hippocampal sparing using RA technique. This can be useful because skilled users need several hours to perform image fusion, to contour structures and to develop a treatment plan, which will need another hour for physics quality assurance. So knowing the feasibility of sparing hippocampus according to the site or the diameter of lesion, everyone could decide to use VMAT-RA techniques or not without spending many hours to obtain only a little sparing. Our data show that when the lesion is in temporal lobe, near the hippocampus, the feasibility of minimizing the mean hippocampal dose is lower. Ghia et al. [11] demonstrated that hippocampus can be spared because metastases are generally more than 5 mm away. According our method the temporal lesion with 2 and 3 cm in diameter are partially located into the hippocampus, while if the lesion is of 1 cm of diameter, the distance from lateral side of hippocampus is of 0.5 cm. 

On the contrary, sparing hippocampus is feasible when brain lesions are in other regions, also if close to critical structures far from hippocampus. Metastases located in occipital lobe show the lowest mean hippocampal dose. The impact of size of brain lesions is lower on hippocampal dose. 

Regarding hippocampus dose with helical tomotherapy (HT) technique, Gutiérrez et al. [16] showed the possibility to reduce the dose to the hippocampus until 6 Gy^2^ treating the whole brain to a *D*95% of 32.25 Gy with SIB technique, while Marsh et al. [17] delivered 30 or 35 Gy in 15 or 14 fractions on whole brain obtaining a mean dose/equivalent uniform dose (EUD) at hippocampus of 12.5/14.23 Gy. 

Gondi et al. [12] observed that HT spared hippocampus more than LINAC based IMRT, while target coverage and homogeneity were acceptable with both technologies. 

Other researches tested VMAT or compared this technology to tomotherapy. Lagerwaard et al. [18] tested the efficacy of RA treatment planning to deliver WBRT with SIB in patients with multiple brain metastases, without sparing the hippocampal region, and it showed excellent coverage of planning target volume for WBRT and metastases.

Hsu et al. [14] found that VMAT was able to deliver to metastases a radiosurgical dose during WBRT. For the whole brain, the mean target coverage and homogeneity index were 0.960 ± 0.002 and 0.39 ± 0.06, respectively. The mean hippocampal dose was 5.23 ± 0.39 Gy^2^; he used Varian Eclipse external Beam Planning System, version 7.1, and a pencil beam algorithm. 

Prokic et al. [19] recently observed that for patients with up to 8 metastases the SIB is more effective than the WBRT + stereotactic fractionated irradiation in lowering doses to the hippocampus. In the SIB schedule, the prescribed dose was 30 Gy in 12 fraction to the WB and 51 Gy in 12 fraction to individual brain metastases. The mean dose to the hippocampus ranged from 7.55 ± 0.62 to 6.29 ± 0.62; he used Eclipse version 10.0 with an optimization system PRO3.

We obtained a mean hippocampal dose ranged from 8.90 Gy^2^ to 47.16 Gy^2^. In the most favourable situation, such as metastasis in the occipital lobe or diameter of 1 cm, mean hippocampal doses were 9.68 Gy^2^ and 9.45 Gy^2^ respectively. These data are better than those reported by Marsh et al. [17] but worse than those obtained by other authors. 

The gap could be related more to algorithm for dose's optimization than to that of dose's calculation because in the brain the different accuracy between the pencil beam and the AAA algorithm could not be significant. 

Regarding HI of whole brain, we found an HI that ranged from 0.34 to 0.9 with a mean of 0.65 ± 0.18; our results are more similar to those reported by Gutiérrez et al. [16] with tomotherapy (0.485 ± 0.152), comparable with those obtained by Hsu et al. [14] with VMAT (0.39 ± 0.06), Prokic et al. [19] (0.54 ± 0.04) with VMAT, and by Gondi et al. [12] with LINAC-IMRT; it is worse only them those published by Gondi et al. when used helical tomotherapy (0.008–0.29). However, sparing the hippocampus and concomitantly boosting the metastases deliberately increases the heterogeneity for the whole brain volume for this composite plan. This makes the HI a poor measure. This is observed also by other investigators [20].

## 4. Conclusions

Our predictive model suggests that metastases in temporal lobe does not allow to reduce significantly the dose to hippocampus. Volumetric modulation RA is able to reduce the mean dose per fraction to the hippocampus. Different version or algorithm of VMAT-RA can influence dose's estimation to hippocampus. Further investigations are necessary to improve statistical analysis and to meet definitive considerations, modelling multiple metastasis, scanning imaging and fusion of other patients, or evaluating hippocampus as both single and paired structures.

## Figures and Tables

**Table 1:** Parameters of 15 RA plans.

Plan	Diameter (cm)	Mean Hippocampal Dose (Gy)	Mean Hippocampal dose (Gy^2^)	HI-PTV whole brain	TC-PTV metastases	TC-PTV whole brain
DS 1	1	12.55	8.90	0.34	0.52	0.82
F 1	1	13.95	10.22	0.36	0.51	0.92
O 1	1	12.82	9.15	0.64	0.54	0.99
P 1	1	13.25	9.55	0.47	0.54	0.91
T 1	1	36.10	33.9	0.55	0.53	0.96
DS 2	2	13.46	9.75	0.78	0.55	0.92
F 2	2	13.49	9.78	0.78	0.72	0.90
O 2	2	13.68	9.96	0.53	0.52	0.92
P 2	2	14.87	11.12	0.57	0.54	0.93
T 2	2	42.00	40.27	0.68	0.48	0.90
DS 3	3	17.80	14.18	0.77	0.49	0.93
F 3	3	15.45	11.70	0.90	0.42	0.91
O 3	3	13.66	9.94	0.60	0.51	0.93
P 3	3	14.76	11.01	0.84	0.46	0.93
T 3	3	50.4	47.16	0.85	0.52	0.80

DS: deep structure; F: frontal lobe; O: occipital lobe; P: parietal lobe; T: temporal lobe; 1–3: metastases diameter (cm).
